# Human Leukocyte Antigen (HLA) System: Genetics and Association with Bacterial and Viral Infections

**DOI:** 10.1155/2022/9710376

**Published:** 2022-05-26

**Authors:** Sadeep Medhasi, Narisara Chantratita

**Affiliations:** Department of Microbiology and Immunology, Faculty of Tropical Medicine, Mahidol University, Bangkok, Thailand

## Abstract

The human leukocyte antigen (HLA) system is one of the most crucial host factors influencing disease progression in bacterial and viral infections. This review provides the basic concepts of the structure and function of HLA molecules in humans. Here, we highlight the main findings on the associations between HLA class I and class II alleles and susceptibility to important infectious diseases such as tuberculosis, leprosy, melioidosis, *Staphylococcus aureus* infection, human immunodeficiency virus infection, coronavirus disease 2019, hepatitis B, and hepatitis C in populations worldwide. Finally, we discuss challenges in HLA typing to predict disease outcomes in clinical implementation. Evaluation of the impact of HLA variants on the outcome of bacterial and viral infections would improve the understanding of pathogenesis and identify those at risk from infectious diseases in distinct populations and may improve the individual treatment.

## 1. Introduction

The cell-mediated adaptive immune response is regulated by the major histocompatibility complex (MHC) or human leukocyte antigen (HLA) in humans [1]. HLA molecules are cell surface glycoproteins whose primary function is to present endogenous and exogenous antigens to T lymphocytes for recognition and response [2]. The HLA molecules that present antigen to T lymphocytes are divided into two main classes: HLA class I and HLA class II molecules. HLA class I molecules play an essential role in the immune defense against intracellular pathogens, whereas HLA class II molecules are predominantly involved in displaying peptides from extracellular pathogens [3]. The HLA region is highly polymorphic, and polymorphisms in the HLA molecules result in variability in amino acid sequences of HLA molecules and thus affect the peptide binding specificity [4]. HLA molecules encoded by different alleles have different peptide-binding repertoires [5]. The polymorphisms in the HLA locus contribute to the genetic diversity of humans and the differences in susceptibility to diseases among genetically distinct groups, thus offering evolutionary advantages of a diverse immunological response to a wide range of infectious pathogens [6]. The associations between HLA alleles and susceptibility to or protection from infectious diseases have been well documented. However, the molecular mechanism underlying host HLA function to infection remains far from understood. Infectious disease continues to affect poor and marginalized populations; therefore, it is essential to utilize the increasing knowledge and technological advances in HLA typing to study the pathogenesis and development of novel therapeutic targets in infectious diseases of public health concerns.

Genetic variations at the loci encoding HLA genes are associated with susceptibility or protection to infectious diseases. Genetic studies have found an association between the HLA alleles or haplotypes and bacterial infectious diseases, including tuberculosis, leprosy, and melioidosis [7–9]. Identifying risk and protective HLA alleles will provide critical insights into the mechanisms that influence the pathogenesis of infections and protection. HLA typing can identify associations between HLA alleles and infections in an individual [10]. Patients exhibit different immune responses to bacterial and viral infections, and HLA molecules play an essential role in regulating the host's immune response. Therefore, the reported HLA alleles contributing to the susceptibility or protective effect to bacterial and viral infections will aid in elucidating the immunological mechanisms in disease outcomes [11, 12].

This review will provide the basic concepts of HLA and the current status of the HLA associations with bacterial and viral infections across world populations. This review primarily focuses on predisposing risk and protective HLA alleles among several populations in major infectious diseases, including bacterial infections (tuberculosis, leprosy, melioidosis, and *Staphylococcus aureus* infections) and viral infections (human immunodeficiency virus (HIV) infection, coronavirus disease 2019 (COVID-19), hepatitis B, and hepatitis C). Many studies on these infections have shown HLA-associated susceptibility in many populations, but the association with melioidosis and *S. aureus* infections is less characterized. We also discuss the challenges of complicating disease outcome prediction through HLA typing. A deeper understanding of the genetic basis of susceptibility to these infections will aid in understanding the pathogenesis of the disease, identify new molecular targets for prophylactic and therapeutic interventions, and develop a potential tool to identify those at risk of rapid disease progression.

## 2. Structure and Function of Human Leukocyte Antigen

**Figure 1:**
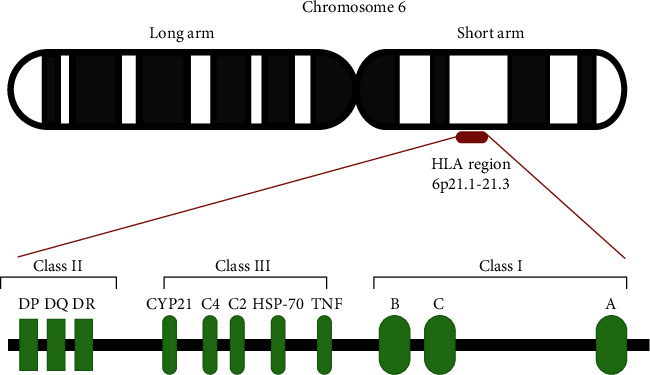
Schematic representation of the HLA locus on human chromosome 6.

The HLA molecule is the name for the human MHC, which orchestrates immune regulation by antigen presentation to T cells [13]. The HLA system resides in a region that spans approximately 4,000 kilobases (kb) of DNA on the short arm of chromosome 6 (6p21). This region encodes three major classes of proteins, HLA class I (HLA-A, HLA-B, and HLA-C), class II (HLA-DP, HLA-DQ, and HLA-DR), and class III (components of the complement system, 21-hydroxylase, heat shock protein, and tumor necrosis factors) (Figure 1) [14, 15].

**Figure 2:**
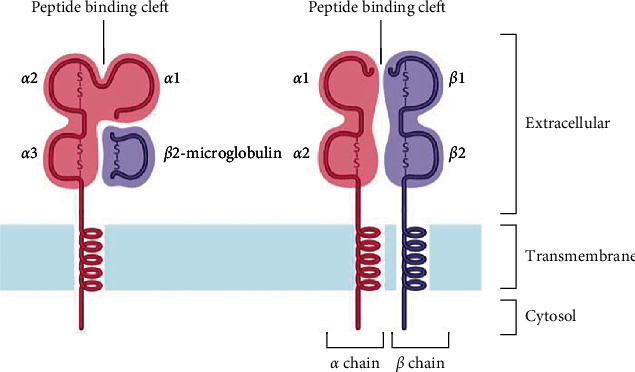
Schematic presentation of the structure of HLA class I (a) and class II (b) molecules.

HLA class I molecules are present as transmembrane glycoproteins on the surface of nearly all nucleated cells. These molecules present intracellular self- or non-self-antigens to CD8^+^ cytotoxic T cell receptors and killer cell immunoglobulin-like receptors (KIR) [16]. HLA class I molecules consist of two heterodimer polypeptide chains, a heavy *α* chain, and a lighter *β*2-microglobulin chain. The *α* chain has three extracellular domains (*α*1, *α*2, and *α*3), a transmembrane region, and a C-terminal cytoplasmic tail. The two domains, *α*1 and *α*2, fold to form a peptide-binding groove and are referred to as the recognition region. The *β*2-microglobulin chain is primarily associated with the *α*3 domain and is responsible for HLA stability (Figure 2(a)) [17, 18].

HLA class II molecules are present on the surface of antigen-presenting cells (APC), such as macrophages, B cells, and dendritic cells, and display short antigen peptides to CD4^+^ helper T cells and their receptors [19]. HLA class II molecules consist of two polypeptide chains (an *α* and a *β* chain), and each chain is folded into two separate domains: *α*1 and *α*2 and *β*1 and *β*2, respectively. A peptide-binding groove is formed by the distal *α*1 and *β*1 domains. The proximal domains, *α*2 and *β*2, are highly conserved to which the T cell receptor (TCR) binds (Figure 2(b)) [18].

Unlike HLA class I and HLA class II regions, whose functions in the immune response are well defined, the HLA class III region encodes for various inflammatory molecules, complement, and heat shock protein [11]. The HLA class III region spans 700 kb of DNA and is located between the centromeric class II (*HLA-DRA*) and the telomeric class I regions (*MICB*) (Figure 1) [20].

## 3. HLA Nomenclature

**Figure 3:**
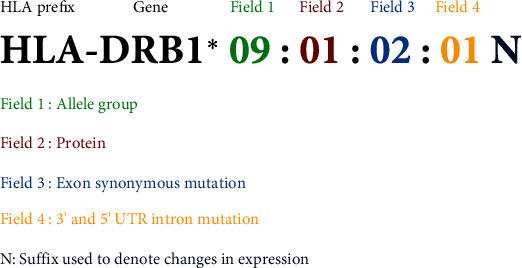
HLA molecule nomenclature with information between prefix (HLA) and suffix (N).

The WHO Nomenclature Committee for Factors of the HLA System is responsible for the formal naming of HLA alleles and has reported the names through two websites, Immuno Polymorphism Database-International ImMunoGeneTics project/HLA (IPD-IMGT/HLA) database (https://www.ebi.ac.uk/ipd/imgt/hla/) and HLA Nomenclature (http://hla.alleles.org/nomenclature/naming.html) [21, 22]. The current HLA nomenclature system uses a unique number corresponding to up to four sets of digits separated by colons (Figure 3). The HLA-prefix signifies the human MHC gene complex. The next portion after the HLA-prefix indicates the specific HLA genomic region. The first two digits of the number (field 1) show the allele group (or allele family). The second field provides the specific HLA allele (HLA protein). The third field names the alleles that differ only by synonymous nucleotide substitutions within the coding region. The fourth field names the alleles that vary only by sequence polymorphisms in introns, 3′-untranslated regions, and 5′-untranslated regions. Last is the suffix consisting of a letter that denotes alleles with changes in expression levels of the HLA protein products. The suffix “N” is used for null alleles with no HLA protein expression. Other letters have been used to designate an allele to indicate its expression status: L: low expression, S: secreted, and Q: questionable. A standardized HLA nomenclature has contributed to the understanding of the HLA system and proved to be an essential resource to address HLA typing ambiguity in the clinical applications of HLA [23].

## 4. Genetic Association between HLA Loci and Infectious Diseases

The HLA family of genes is one of the most polymorphic genes in the human genome [24]. The IPD-IMGT/HLA Database is a repository for the variant sequences of HLA alleles. As of April 2022, the IPD-IMGT/HLA Database has reported 33,490 HLA alleles. Of the 24,308 HLA class I alleles, 7,452, 8,849, and 7,393 alleles are counted in HLA-A, HLA-B, and HLA-C genes. Of 9,182 HLA class II alleles, 32, 4,018, 442, 40, 2,230, 406, 5, 1,958, and 6 alleles are counted in HLA-DRA, HLA-DRB, HLA-DQA1, HLA-DQA2, HLA-DQB1, HLA-DPA1, HLA-DPA2, HLA-DPB1, and HLA-DPB2 genes, respectively (https://www.ebi.ac.uk/ipd/imgt/hla/about/statistics/) (Table 1) [21]. Research in infectious diseases has not described the strongest association with HLA class III in different ethnic groups. Our review will assess the association studies focusing on HLA class I and class II variants associated with susceptibility or protection to infectious diseases.

### 4.1. HLA Associations with Tuberculosis

Tuberculosis (TB), caused by *Mycobacterium tuberculosis* (*M*. *tuberculosis*), is an infectious disease posing a significant public health threat primarily in low- and middle-income countries [25]. The World Health Organization reported an estimated 10.0 million TB cases, 1.2 million TB deaths among HIV-negative people, and an additional 208,000 TB deaths among HIV-positive people in 2019 [26]. *M. tuberculosis* can modulate the HLA class II pathway by inhibiting phagosome maturation and thus preventing the formation of bacterial peptide-MHC-II (HLA class II) complexes and subsequent T cell responses to bacterial antigens [27]. *M*. *tuberculosis* also inhibits MHC-II expression and antigen processing resulting in decreased recognition by T cells [28].

Several genetic polymorphisms of HLA have been implicated in individuals' genetic susceptibility to tuberculosis in distinct populations (Table 2). A study on 31 pulmonary tuberculosis patients in Poland showed a higher frequency of *HLA-DRB1*∗*16* in patients when compared to the 58 healthy controls. In comparison, the frequency of the *HLA-DRB1*∗*13* allele was significantly lower in the patient group than in the healthy controls [29]. In Iranian patients with pulmonary tuberculosis, *HLA-DRB1*∗*07* and *HLA-DQA1*∗*01:01* alleles appeared to be the risk alleles, and *HLA-DQA1*∗*03:01* and *HLA-DQA1*∗*05:01* alleles were the protective alleles [30]. Wamala et al. investigated HLA class II gene polymorphisms in susceptibility to pulmonary tuberculosis in Uganda and observed that the *HLA-DQB1*∗*03:03* allele was associated with resistance to pulmonary tuberculosis [31]. In the study performed in South India, the frequencies of *HLA-DRB1*∗*15:01* and *HLA-DQB1*∗*06:01* alleles were higher in pulmonary tuberculosis patients than in the control group. In contrast, the frequency of the *HLA-DPB1*∗*04* allele was highly prevalent among the control group and was deemed to be a protective allele against pulmonary tuberculosis [32]. A study by Sveinbjornsson et al. in Icelanders demonstrated *HLA-DQA1*∗*03* (represented by p.Ala210Thr) and a noncoding variant, rs557011, located between *HLA-DQA1* and *HLA-DRB1* contributing to genetic susceptibility to tuberculosis [33]. They also demonstrated the association of rs9271378 with a reduced risk of pulmonary TB in Icelanders. A first genome-wide association study (GWAS) on tuberculosis in Han Chinese revealed HLA loci, rs41553512 (a missense mutation in *HLA-DRB5*), significantly associated with tuberculosis [34]. Strain-based association analysis between HLA class II genes and tuberculosis in the Thai population identified a significant association of *HLA-DRB1*∗*09:01* and *HLA-DQB1*∗*03:03* with a modern strain of *M*. *tuberculosis* (absence of *M. tuberculosis*-specific deletion 1 (TbD1) region) [35].

### 4.2. HLA Associations with Leprosy

Leprosy is a chronic infectious disease caused by *Mycobacterium leprae*. Based on clinical, histopathological, microbiological, and immunological features, Ridley and Jopling classified the leprosy spectrum into five groups: tuberculoid (TT), borderline-tuberculoid (BT), borderline-borderline (BB), borderline-lepromatous (BL), and lepromatous (LL) [36].

Both HLA class I and class II genes have been implicated in susceptibility to leprosy and its subtypes in different populations (Table 3). In a study in India, the frequencies of *HLA-A*∗*02:06*, *HLA-A*∗*11:02*, *HLA-B*∗*40:16*, *HLA-B*∗*51:10*, *HLA-Cw*∗*04:07*, and *HLA-Cw*∗*07:03* alleles were significantly higher in leprosy patients compared to healthy controls, while the frequencies of *HLA-A*∗*0101*, *HLA-Cw*∗*04011*, and *HLA-Cw*^∗^*06:02* alleles were markedly lower in leprosy patients compared to healthy controls [37]. Another study of genetic susceptibility to leprosy in India found rs1071630 located in *HLA-DQA1* and rs9270650 in *HLA-DRB1* associated with susceptibility to leprosy [38]. A genome-wide association study in 706 patients with leprosy and 1225 unaffected controls in Han Chinese found a single-nucleotide polymorphism (SNP) rs602875 at the *HLA-DR-DQ* locus associated with susceptibility to leprosy [39]. HLA-DR molecules activate T cells by presenting *M. leprae* peptide antigens to CD4^+^ T cells and activate various pathways. Anomalies in those pathways could cause HLA-associated leprosy [39]. The *HLA-DRB1*∗*15* allele was associated with leprosy, while *HLA-DRB1*∗*09* was significantly protective against leprosy in Han Chinese [40]. A meta-analysis by Zhang et al. identified *HLA-DQA1*∗*03:03* and *HLA-C*∗*08:01* as causal variants to leprosy susceptibility in the Han Chinese population [41]. In the association study between *HLA-DRB1* and leprosy among Brazilian and Vietnamese people, the *HLA-DRB1*∗*04* allele was associated with protection against leprosy, and the *HLA-DRB1*∗*10* allele was found to be associated with susceptibility to leprosy [42]. A recent study was conducted to investigate the association of HLA class I and II genes with leprosy in a Brazilian population. The study identified the association of *HLA-C*∗*12* and *HLA*-*DPB1*∗*105* with susceptibility to leprosy, while *HLA-C*∗*08*, *HLA-DPB1*∗*04*, and *HLA-DPB1*∗*18* were protective against leprosy [43]. Dallmann-Sauer et al. performed next-generation sequencing to genotype three HLA class I and eight class II genes in 1,155 individuals from a Vietnamese leprosy case-control sample. The *HLA-DQA1*∗*01:05* and *HLA-DRB1*∗*10:01* alleles in complete linkage disequilibrium (LD) were associated with leprosy, whereas the *HLA-C*∗*07:06* allele was shown to be protective against leprosy in the Vietnamese population [44]. A study of the association of *HLA-DRB1* alleles in 71 leprosy patients and 81 healthy controls in Argentina found a higher frequency of *HLA-DRB1*∗*14:01* and *HLA-DRB1*∗*14:06* alleles in leprosy patients compared to controls. In contrast, the frequency of *HLA-DRB1*∗*08:08* and *HLA-DRB1*∗*11:03* was highly prevalent among the healthy controls compared to the leprosy patients hence indicating resistance to leprosy [45]. Interestingly, a study in Taiwan assessing the leprosy association with HLA class I and class II alleles found a protective effect of *HLA-DRB1*∗*04:05* on multibacillary leprosy [46].

### 4.3. HLA Associations with Melioidosis

Melioidosis is an infectious disease caused by the Gram-negative bacillus *Burkholderia pseudomallei*. Melioidosis is widely endemic in Southeast Asia, especially in Thailand, and northern Australia [47]. The disease is highly seasonal, and the organism is commonly found in soil and water in the endemic areas [48]. Risk factors include diabetes mellitus, chronic kidney disease, chronic lung disease, alcohol abuse, and steroid therapy [49, 50]. Diabetes mellitus is the major underlying risk factor occurring in 60-75% of patients diagnosed with melioidosis [50–52]. Clues to the mechanisms involved between diabetes mellitus and melioidosis might be explained by the role of HLA alleles in both diabetes mellitus and melioidosis. HLA class II alleles have been documented to have prominent effects on diabetes mellitus in distinct populations [53–57].

Studies in Thailand have reported the risk of HLA alleles associated with melioidosis (Table 4). In 1998, Dharakul et al. investigated the associations between HLA class II alleles and melioidosis in 79 melioidosis patients and 105 healthy controls in Northeast Thailand [8]. The study demonstrated a significant association between the *DRB1*∗*16:02* allele and the susceptibility to melioidosis in the Thai population. In addition, associations were observed with the *DRB1*∗*16:02* allele for severe melioidosis and septicemic melioidosis when various clinical groups of melioidosis patients were compared with healthy controls. In another study in Northeast Thailand, *HLA-B*∗*46* and *HLA-C*∗*01* (HLA class I alleles) were associated with increased mortality from acute melioidosis compared to the survived patients from acute melioidosis [58].

In melioidosis, suppressed HLA-DR expression on classical monocytes was associated with poor outcomes [59]. A transcriptomic analysis of changes in gene expression of nonsurvivors from melioidosis in Northeast Thailand found the downregulation of HLA class II genes, including *HLA-DPB1*, H*LA-DRA*, *HLA-DOA*, and *HLA-DOB* [60]. The role of HLA in the pathogenesis and poor prognosis of melioidosis is not fully understood. Reynolds et al. demonstrated a strong binding affinity of alkyl hydroperoxide reductase (AhpC), a highly dominant *B. pseudomallei* antigen, with *HLA-DR* alleles, *HLA-DR1*, *HLA*-*DR3*, *HLA-DR4*, *HLA-DR7*, *HLA-DR9*, *HLA-DR11*, *HLA-DR13*, *HLA-DR15:01*, and *HLA-DR15:02*, and the *HLA-DQ* alleles, *HLA*-*DQB1*∗*06:02* and *HLA*-*DQB1*∗*03:02* [61]. In addition, the study also reported that among patients with acute melioidosis in Northeast Thailand, survival was associated with a strong HLA class II-restricted T cell response to AhpC.

### 4.4. HLA Associations with *Staphylococcus aureus* Infections

*S. aureus* is an opportunistic pathogen and a leading cause of morbidity and mortality in hospital and community settings [62]. The *S*. *aureus* superantigens, toxic shock syndrome toxin-1 (TSST-1) and *S. aureus* enterotoxin B (SEB), bind to the HLA class II molecule HLA-DR1 [63]. Genetic variations within the host are associated with susceptibility to *S. aureus* infections suggesting why one-third of humans are known to be colonized with *S. aureus* in their anterior nares, but most avoid clinically significant *S. aureus* infections [64].

Several HLA alleles are proposed as susceptibility factors to *S*. *aureus* infection (Table 5). A GWAS conducted to identify specific genetic variants that underlie susceptibility to infections caused by *S*. *aureus* in white subjects reported three SNPs, rs4321864 located in the *HLA-DRA* gene and rs115231074 and rs35079132 located in *HLA-DRB1* genes, associated with *S*. *aureus* infection. The study also found an association between *HLA-DRB1*∗*04* serotype and *S*. *aureus* infection [65]. Cyr et al. evaluated the role of genetic variation on susceptibility to *S*. *aureus* bacteremia in African Americans. They found the genetic association of one region on chromosome 6 in the HLA class II region with susceptibility *S*. *aureus* bacteremia [66].

### 4.5. HLA Associations with HIV Infection

HIV infection is a major global public health issue with a devastating impact on social and economic indicators [67–69]. HLAs play a complex role in immunomodulation during HIV infection, and variations at the HLA class I locus have been linked to the efficiency of CD8^+^ T cell control of viremia [70].

Polymorphisms within HLA class I and II loci have been identified as the host genetic modifier of HIV disease progression in several populations (Table 6). In the Argentinian population, the frequency of the *HLA-B*∗*39* allele was significantly higher in HIV-1-positive subjects than in controls, whereas the *HLA-B*∗*44* allele was absent among the HIV-1-positive subjects [71]. Claiborne et al. identified four HLA class I alleles (*B*∗*14:01*, *B*∗*57*, *B*∗*58:01*, and *B*∗*81*) and two HLA class II alleles (*DQB1*∗*02* and *DRB1*∗*15*) associated with the protection from rapid CD4^+^ T cell decline without controlling early plasma viral load in a Zambian early infection cohort [72]. A GWAS in HIV-1 infected Caucasian subjects showed *HLA-B*∗*57:01* rs2395029 and *HLA-C* rs9264942 associated with HIV-1 disease progression [73]. Analysis of the HLA-B allele frequencies among HIV-1-infected individuals classified as rapid progressors (RPs), typical progressors (TPs), and long-term nonprogressors (LTNPs) in the Brazilian population revealed the higher frequency of the *HLA-B*∗*52* allele in the LTNP group than in either the TP group or the RP group, and thus, the presence of the *HLA-B*∗*52* allele is favorable to slow AIDS progression [74]. A study involving treatment-naive patients with chronic HIV-1 infection from (i) Warsaw, Poland; (ii) Athens, Greece; (iii) Mexico City, Mexico; (iv) Bonn, Germany; (v) Boston, MA; (vi) Barcelona, Spain; and (vii) Thames Valley, UK, suggested that *HLA-B*∗*27:02* was associated with slower progression to HIV disease [75]. In a study among the HIV clade B-infected ART-naïve individuals from Mexico and Central America, several HLA alleles were identified as protective (*A*∗*03:01*, *B*∗*15:39*, *B*∗*27:05*, *B*∗*39:02*, *B*∗*57:01/02/03*, and *B*∗*58:01*) and risk (*A*∗*68:03/05*, *B*∗*15:30*, *B*∗*35:02*, *B*∗*35:12/14*, *B*∗*39:01/06*, *B*∗*39:05*, and *B*∗*40:01*) factors for disease progression [76].

### 4.6. HLA Associations with COVID-19

COVID-19 is caused by the severe acute respiratory syndrome coronavirus 2 (SARS-CoV-2). It is a worldwide pandemic with 198,778,175 confirmed cases, including 4,235,559 deaths (as of 3 August 2021) [77]. Patients with severe COVID-19 have been found to exhibit immune dysregulation characterized by IL-6-mediated low HLA-DR expression [78].

Several HLA polymorphisms are associated with susceptibility and severity to COVID-19 in different populations (Table 7). In a study among individuals of European descent experiencing variable clinical outcomes following COVID-19 infection, the frequency of *HLA-DRB1*∗*04:01* was higher among asymptomatic COVID-19 patients than the severe COVID-19 patients and suggested the protective effects of the *HLA-DRB1*∗*04:01* allele against developing severe complications from COVID-19 [79]. The *DRB1*∗*09:01* allele was associated with risk for severe COVID-19 in Japanese [80]. Novelli et al. analyzed the HLA allele frequency distribution in Italian COVID-19 patients to identify potential markers of susceptibility to the disease and observed that the frequencies of *HLA-DRB1*∗*15:01*, *HLA-DQB1*∗*06:02*, and *HLA-B*∗*27:07* alleles were higher among the severe affected COVID-19 patients compared to healthy controls [81]. In a comprehensive *in silico* analysis, HLA-B∗46:01 had the fewest predicted binding peptides for SARS-CoV-2, indicating that the individuals with this allele may be particularly vulnerable to COVID-19 [82]. Another *in silico* analysis found the association between *HLA-A*∗*02:01* and an increased risk for COVID-19, and *HLA-A*∗*02:01* was predicted to present the lower SARS-CoV-2 antigens and subsequent lower T cell-mediated antiviral responses compared to *HLA-A*∗*11:01* or *HLA-A*∗*24:02* alleles [83]. In a study conducted by Shkurnikov et al. in deceased patients with COVID-19 in Russia, *HLA-A*∗*01:01* was associated with early COVID-19 deaths among the high-risk patients, and *HLA-A*∗*02:01* and *HLA-A*∗*03:01* alleles were associated with early COVID-19 deaths among the low-risk patients [84].

### 4.7. HLA Associations with Hepatitis B

Hepatitis B is a significant public health problem putting people at high risk of death from cirrhosis and liver cancer [85]. Hepatitis B is caused by the hepatitis B virus (HBV). HBV-specific CD8^+^ cytotoxic T lymphocytes play a critical role in viral clearance and liver injury, and HLA polymorphisms have been reported to alter CD8^+^ cytotoxic T lymphocyte responses [86].

Multiple population association studies have provided evidence of an association between HLA locus variations and hepatitis B virus infection (Table 8). A study comparing the distribution of HLA alleles between persistent and transient HBV infection in children and adults in the Gambia found *HLA-DRB1*∗*13:02* associated with protection against persistent HBV infection among children and adults [87]. A Chinese study by Fan et al. showed an association between the *HLA-DQ* rs9275319C allele and decreased HBV infection risk and an increased HBV clearance [88]. Another Chinese study showed that *HLA-DQB1*∗*06:03* protected against HBV infection [89]. An association analysis performed among the Turkish population revealed the association of the *HLA-DPB1* rs9277535A allele with the risk of persistent HBV infection [90]. A Caucasian study showed that *HLA-A*∗*03:01* was associated with viral clearance, and *HLA-B*∗*8* was associated with viral persistence [91]. Al-Qahtani et al. demonstrated an association of *HLA-DQ* alleles (rs2856718A and rs9275572A) and *HLA-DP* alleles (rs3077G and rs9277535G) with HBV infection in Saudi Arabian patients [92]. Nishida et al. showed that *HLA-DQB1*∗*06:01* was associated with chronic HBV infection in Japanese patients [93].

### 4.8. HLA Associations with Hepatitis C

Hepatitis C virus (HCV) infection is a significant cause of acute and chronic hepatitis. Chronic hepatitis leads to liver cirrhosis and hepatocellular carcinoma (HCC) [94, 95]. HCV persistence or clearance is proposed to depend on the response of the HLA class I-restricted HCV-specific CD8^+^ cytotoxic T cell-mediated lysis of virus-infected host cells [96].

Depending on ethnicity, a significant association has been suggested between HLA alleles and HCV persistence or spontaneous clearance (Table 9). In the Thai population, the frequency of *HLA- DRB1*∗*03:01* and *HLA-DQB1*∗*02:01* was higher in the persistent HCV infection group than in the transient HCV infection group, revealing their susceptibility effect on persistent HCV infection [97]. Genotyping a large multiracial cohort of US women to evaluate associations between HLA alleles and HCV viremia indicated some HLA alleles (*B*∗*57:01*, *B*∗*57:03*, *Cw*∗*01:02*, and *DRB1*∗*01:01*) were associated with the absence of HCV RNA. At the same time, the presence of HCV RNA was observed for *HLA-DRB1*∗*03:01* [98]. Huang et al. reported the association of *HLA-A*∗*02:01* and *HLA-DRB1*∗*11:01* with HCV spontaneous clearance in the Chinese population [99]. In Egyptian HCV patients and their families or close household contacts, HLA-DRB1 allele associations with HCV were reported (*DRB1*∗*03:01:01* and *DRB1*∗*13:01:01* alleles and the risk of progression to chronic hepatitis C infection and *DRB1*∗*04:01:01*, *DRB1*∗*04:05:01*, *DRB1*∗*07:01:01*, and *DRB1*∗*11:01:01* and protection against HCV infection) [100].

## 5. Challenges in HLA Typing to Predict Disease Outcomes

### 5.1. HLA Diversity

The genetic diversity of HLA within each population can be explained or measured by allelic richness (ar) and the expected heterozygosity (*H*). The allelic richness of a population at a particular locus is the expected number of alleles present in the population at that locus [101]. The expected heterozygosity is defined as the average proportion of heterozygotes per locus in a randomly mating population [102]. Pathogen richness is the number of pathogens within a defined geographical region [103]. Sanchez-Mazas et al. reported a significant positive correlation between genetic diversity and pathogen richness at *HLA-A* and *HLA-B* and a significant negative correlation at *HLA-DQB1* [104].

Identifying the most clinically relevant HLA variant is necessary for facilitating improvements in the diagnosis and treatment of human disease. The identifiable HLA variants provide opportunities to refine medical management to optimize patient health and medical outcomes. However, genetic diversity within and between populations poses a challenge in HLA genomics to become a standard component of health care. Many rare HLA variants are identified, and they are likely to contribute to interindividual differences in risk or protection to disease. HLA data from various populations have been collected, but populations of African and Asian descent have limited representation to provide insight into HLA disease associations [105]. HLA genetic diversity among Europeans is well documented [106–108]. Hurley et al. recently reported global frequencies of common, intermediate, and well-documented HLA alleles and highlighted the HLA diversity in world populations [109].

### 5.2. HLA Genotyping

Different DNA-based molecular techniques represent the modern methods used for HLA typing in clinical applications. Depending on their power to discriminate between HLA alleles, DNA-based HLA typing methods are characterized by low resolution (result at the level of the digits composing the first field in the HLA nomenclature) and high resolution (result at the level of four digits) [110]. The most widely used DNA-based methods in conjunction with PCR for HLA typing include sequence-specific oligonucleotides (SSO), sequence-specific primers (SSP), and sequence-based typing (SBT) [111]. In PCR-SSO, PCR products are hybridized into sequence-specific oligonucleotide probes. In PCR-SSP, primers complementary to particular HLA allele sequences are used, and amplification with sequence-specific primers yields only a product if the target sequences are present in the DNA sample. In SBT, HLA genetic regions are amplified by PCR using locus-specific primers, followed by direct sequencing of the PCR products [112].

Although SSO and SSP methods are widely used, they are not practical and capable of detecting all known HLA polymorphisms and novel HLA alleles [113]. SSO and SSP typing methods struggle to resolve the major allele groups [114]. The SBT provides high-resolution HLA genotyping and can identify new alleles. While SBT allows for a detailed interpretation of HLA alleles, it has limitations, including time-consuming protocols, low throughput, and ambiguities in HLA typing results [115–117]. SNP-based HLA typing on microarray produces a high-resolution HLA type but has not been used in clinical typing due to its tendency to miss several HLA variants [117].

Next-generation sequencing- (NGS-) based HLA typing methods allow high-throughput sequencing, massively parallel analysis, and high-resolution HLA typing with minimal ambiguity [118, 119]. NGS-based HLA typing has been implemented with better accuracy compared to traditional HLA typing methods [120]. NGS-based HLA typing approaches are promising but are not yet ready to be implemented in routine clinical care settings due to the higher cost and complex protocol [111].

### 5.3. Implementing Therapeutic Approaches Using Genomic Knowledge of Specific Targets and Their Roles in Disease

Utilization of HLA typing can identify alleles associated with disease risks and improve clinical outcomes. However, genomic literacy among clinicians is a low to moderate level which presents a challenge in adopting genomic services by clinicians in clinical practices [121]. To overcome this challenge, high-quality results in HLA association studies must be disseminated among the health workforce, including the policymakers and the personnel on the ground. Awareness about the implications of HLA typing into mainstream clinical practice must be raised by educating the health workforce. There is a need for more reporting on the clinical validity and clinical utility of genetic testing used for screening of risk and protective HLA alleles in diseases [122, 123]. Integrating genomic services utilizing HLA-specific tests face challenges and barriers to widespread adoption. These include the lack of a single standard approach to achieve HLA typing by NGS data, integrating electronic health records (EHR) of genomic results and clinical decision support (CDS), ensuring confidentiality for patients and families and lack of reimbursement [124–127].

## 6. Conclusions

This article summarized the findings from association studies of HLA variants with bacterial and viral infections. It is important to note that there were no overlaps in the HLA variants associated with susceptibility or protection to infectious diseases in populations worldwide. Despite the evidence of association of HLA variants with disease susceptibility in our review, a consistent genetic HLA locus has not been demonstrated within the population.

The global pathogens will expand to new geographical locations with genetic mutations in the future [128]. New sequencing techniques that allow faster, cheaper, and less intensive sequencing need to be developed to effectively implement HLA typing in routine clinical care. Advancements in HLA typing technologies are enabling a more accurate linking of HLA genotypes to disease outcomes. NGS technologies will provide a deeper insight into disease mechanisms and biological processes of HLA. Combining HLA sequencing with the expression levels of HLA genes can provide a clearer picture of the role of HLA genes in the pathogenesis of diseases. To understand HLA evolution, HLA expression levels must be integrated with information on HLA genetic variation in diverse populations. At the same time, large data sets need to be generated to reinterpret information to understand pathogen spread in the future [129].

In light of the current evidence, the goal of HLA typing should aid in the realization of “precision medicine” that will benefit patients in diverse populations. Significant research on HLA and infectious diseases is needed in subjects of all ethnic origins to achieve optimum therapeutic outcomes for broader clinical implementation.

## Figures and Tables

**Table 1:** HLA class I and class II genes and number of alleles (April 2022).

HLA locus	Number of alleles	Number of expressed proteins
HLA-A	7,742	4,355
HLA-B	8,849	5,343
HLA-C	7,393	4,095
HLA-DRA	32	5
HLA-DRB	4,018	2,736
HLA-DQA1	442	205
HLA-DQA2	40	11
HLA-DQB1	2,230	1,407
HLA-DPA1	406	173
HLA-DPA2	5	0
HLA-DPB1	1,958	1,223
HLA-DPB2	6	0

**Table 2:** Associations between HLA and tuberculosis.

Population	Study design	Sample size	Serotype, allele, SNP, or haplotype	Type of association	Ref.
Polish	Case-control	31 pulmonary TB patients and 58 healthy controls	*HLA-DRB1*∗*16*	Susceptibility	[29]
			*HLA-DRB1*∗*13*	Protection	
Iranian	Case-control	40 pulmonary TB patients and 100 healthy controls	*HLA-DRB1*∗*07* and *HLA-DQA1*∗*01:01*	Susceptibility	[30]
			*HLA-DQA1*∗*03:01* and *HLA-DQA1*∗*05:01*	Protection	
Uganda	Case-control	43 pulmonary TB patients and 42 healthy controls	*HLA-DQB1*∗*03:03*	Protection	[31]
Indian	Case-control	126 pulmonary TB patients and 87 healthy controls	*HLA-DRB1*∗*15:01* and *HLA-DQB1*∗*06:01*	Susceptibility	[32]
			*HLA-DPB1*∗*04*	Protection	
Icelander	Case-control	3,686 pulmonary TB patients, 14,723 patients with *M. tuberculosis* infection, 8,162 patients with any other forms of TB, and 277,643 healthy controls	rs557011[T] located between *HLA-DQA1* and *HLA-DRB1*	Susceptibility to pulmonary TB and *M. tuberculosis* infection	[33]
			*HLA-DQA1*∗*03*	Susceptibility to *M. tuberculosis* infection	
			rs9271378[G] located between *HLA-DQA1* and *HLA-DRB1*	Reduced risk of pulmonary TB	
Han Chinese	Case-control	4,310 TB patients and 6,386 healthy controls	*HLA-DRB5* rs41553512	Susceptibility	[34]
Thai	Case-control	682 TB patients and 836 healthy controls	*HLA-DRB1*∗*09:01* and *HLA-DQB1*∗*03:03*	Susceptibility to TB caused by modern *M. tuberculosis* strains	[35]

**Table 3:** Associations between HLA and leprosy.

Population	Study design	Sample size	Serotype, allele, SNP, or haplotype	Type of association	Ref.
Indian	Case-control	32 leprosy patients and 67 healthy controls	*HLA-A*∗*02:06*, *HLA-A*∗*11:02*, *HLA-B*∗*40:16*, *HLA-B*∗*51:10*, and *HLA-Cw*∗*04:07*	Susceptibility	[37]
			*HLA-A*∗*0101*, *HLA-Cw*∗*04011*, and *HLA-Cw*∗*06:02*	Protection	
			*HLA-A*∗*11-B*∗*40*, *HLA-A*∗*11:02-B*∗*40:06-Cw*∗*04:07*, and *HLA-A*∗*11:02-B*∗*40:06-Cw*∗*15:02*	Susceptibility to lepromatous leprosy	
Indian	Case-control and family-based	258 leprosy patients, 161 families, and 300 healthy controls	*HLA-DQA1* rs1071630 and *HLA-DRB1* rs9270650	Susceptibility	[38]
Han Chinese	Case-control	3,254 leprosy patients and 5,955 healthy controls	*HLA-DR-DQ* rs602875	Susceptibility	[39]
Han Chinese	Case-control	305 leprosy patients and 527 healthy controls	*HLA-DRB1*∗*15*	Susceptibility	[40]
			*HLA-DRB1*∗*09*	Protection	
Han Chinese	Meta-analysis	Four imputed data sets	*HLA-DQA1*∗*03:03* and *HLA-C*∗*08:01*	Susceptibility	[41]
Brazilian	Case-control	578 leprosy patients and 691 healthy controls	*HLA-DRB1*∗*10*	Susceptibility	[42]
			*HLA-DRB1*∗*04*	Protection	
Brazilian	Case-control	411 leprosy patients and 415 healthy controls	*HLA-C*∗*12* and *HLA*-*DPB1*∗*105*	Susceptibility	[43]
			*HLA-C*∗*08*, *HLA-DPB1*∗*04* and *HLA-DPB1*∗*18*	Protection	
Vietnamese	Family-based	194 families	*HLA-DRB1*∗*10*	Susceptibility	[42]
			*HLA-DRB1*∗*04*	Protection	
Vietnamese	Case-control	687 leprosy patients and 468 healthy controls	*HLA-DQA1*∗*01:05* and *HLA-DRB1*∗*10:01*	Susceptibility	[44]
			*HLA-C*∗*07:06*	Protection	
Argentinean	Case-control	142 leprosy patients and 162 healthy controls	*HLA-DRB1*∗*14 :01* and *HLA-DRB1*∗*14:06*	Susceptibility	[45]
			*HLA-DRB1*∗*08:08* and *HLA-DRB1*∗*11:03*	Protection	
Taiwanese	Case-control	65 multibacillary leprosy patients and 190 healthy controls	*HLA-DRB1*∗*04:05*	Protection against multibacillary leprosy	[46]

**Table 4:** Associations between HLA and melioidosis.

Population	Study design	Sample size	Serotype, allele, SNP, or haplotype	Type of association	Ref.
Thai	Case-control	79 melioidosis patients and 105 healthy controls	*HLA-DRB1*∗*16:02*	Susceptibility and poor prognosis	[8]
Thai	Case-control	183 acute melioidosis patients and 21 healthy controls	*HLA-B*∗*46* and *HLA-C*∗*01*	Increased mortality	[58]

**Table 5:** Associations between HLA and *S. aureus* infections.

Population	Study design	Sample size	Serotype, allele, SNP, or haplotype	Type of association	Ref.
White	Case-control	4,701 culture-confirmed *S. aureus* cases and 45,344 healthy controls	*HLA-DRA*, rs4321864, *HLA-DRB1*, rs115231074 and rs35079132, and *HLA-DRB1*∗*04*	Susceptibility	[65]
African American	Case-control	390 cases and 175 healthy controls	52 SNPs from physical position 32377284 to 32660943 (hg19) in the HLA class II region	Susceptibility	[66]

**Table 6:** Associations between HLA and HIV infection.

Population	Study design	Sample size	Serotype, allele, SNP, or haplotype	Type of association	Ref.
Argentinian	Case-control	56 HIV-1-positive patients and 56 healthy individuals	*HLA-B*∗*39*	Susceptibility	[71]
			*HLA-B*∗*44*	Protection	
Zambian	Longitudinal	127 subjects with acute HIV-1 infections	*HLA*-*B*∗*14:01*, *B*∗*57*, *B*∗*58:01* and *B*∗*81*, and *HLA*-*DQB1*∗*02* and *DRB1*∗*15*	Slow disease progression	[72]
Caucasian	Longitudinal	2,554 HIV-1 infected subjects	*HLA-B*∗*5701*, rs2395029 and *HLA-C*, rs9264942	Accelerated disease progression	[73]
Brazilian	Retrospective observational	218 HIV-1 infected subjects	*HLA-B*∗*52*	Slow disease progression	[74]
Mexican and Central American	Multicenter cross-sectional	3,213 HIV clade B-infected patients	*HLA-A*∗*68:03/05*, *HLA*-*B*∗*15:30*, *B*∗*35:02*, *B*∗*35:12/14*, *B*∗*39:01/06*, *B*∗*39:05*, and *B*∗*40:01*	Accelerated disease progression	[76]
			*HLA-A*∗*03:01*, *HLA-B*∗*15:39*, *B*∗*27:05*, *B*∗*39:02*, *B*∗*57:01/02/03*, and *B*∗*58:01*	Slow disease progression	

**Table 7:** Associations between HLA and COVID-19.

Population	Study design	Sample size	Serotype, allele, SNP, or haplotype	Type of association	Ref.
European	Case-control	49 severe COVID-19 patients and 69 asymptomatic COVID-19 patients	*HLA-DRB1*∗*04:01*	Protection against disease severity	[79]
Japanese	Case-control	73 severe COVID-19 patients and 105 nonsevere COVID-19 patients	*HLA-DRB1*∗*09:01*	Risk of severe COVID-19	[80]
Italian	Case-control	99 severe COVID-19 patients and 1,017 healthy controls	*HLA-B*∗*27:07*, *HLA-DQB1*∗*06:02*, and *HLA-DRB1*∗*15:01*	Risk of severe COVID-19	[81]
Russian	Case-control	111 deceased patients with confirmed COVID-19 and 428 healthy controls	*HLA-A*∗*01:01*, *HLA-A*∗*02:01*, and *HLA-A*∗*03:01*	Early COVID-19 deaths	[84]

**Table 8:** Associations between HLA and hepatitis B.

Population	Study design	Sample size	Serotype, allele, SNP, or haplotype	Type of association	Ref.
Gambian	Case-control	185 children with persistent HBV infection, 218 children with transient HBV infection, 40 adults with persistent infection, and 195 adults with transient HBV infection	*HLA-DRB1*∗*13:02*	Protection against persistent HBV infection	[87]
Chinese	Case-control	397 chronic hepatitis B subjects, 434 HBV spontaneous clearance subjects, and 238 healthy controls	*HLA-DQ*, rs9275319C	Decreased HBV infection risk and an increased HBV clearance	[88]
Chinese	Case-control	256 patients with HBV infection and 433 healthy controls	*HLA-DQB1*∗*06:03*	Protection against chronic HBV infection	[89]
Turkish	Case-control	294 chronic HBV infection patients and 234 persons with HBV natural clearance	*HLA-DPB*, rs9277535A	Risk of persistent HBV infection	[90]
Caucasian	Nested case-control	194 persistent HBV infection individuals and 342 controls with viral clearance	*HLA-A*∗*03:01*	Increased HBV clearance	[91]
			*HLA-B*∗*8*	Risk of persistent HBV infection	
Saudi Arabian	Case-control	488 inactive HBV carriers, 208 active HBV carriers, 85 HBV-infected patients suffering from cirrhosis or cirrhosis and hepatocellular carcinoma, 304 HBV-cleared individuals and 587 healthy uninfected controls	*HLA-DQ*, rs2856718A, and *HLA-DP*, rs3077G, and rs9277535G	Risk of HBV infection	[92]
			*HLA-DQ*, rs9275572A	Protective effect against HBV infection and increased HBV clearance	
Japanese	Case-control	805 HBV patients and 2,278 healthy controls	*HLA-DQB1*∗*06:01*	Risk of chronic HBV infection	[93]

**Table 9:** Associations between HLA and hepatitis C.

Population	Study design	Sample size	Serotype, allele, SNP, or haplotype	Type of association	Ref.
Thai	Case-control	57 subjects with persistent HCV infection and 43 subjects with transient HCV infection	*HLA- DRB1*∗*03:01* and *HLA-DQB1*∗*02:01*	Persistent HCV infection	[97]
			HLA-DRB1∗03:01-HLA-DQA1∗05:01-HLA-DQB1∗02:01	Persistent HCV infection	
Multiracial US women	Case-control	622 HCV RNA positive women and 136 HCV RNA negative women	*HLA-B*∗*57:01*, *B*∗*57:03*, *HLA-Cw*∗*01:02*, and *HLA-DRB1*∗*01:01*	HCV clearance	[98]
			*HLA-DRB1*∗*03:01*	Persistent HCV infection	
Chinese	Case-control	429 subjects with persistent HCV infection and 231 subjects with HCV clearance	*HLA-A*∗*02:01* and *HLA-DRB1*∗*11:01*	HCV clearance	[99]
Egyptian	Family-based and case-control	162 Egyptian families (255 subjects with chronic hepatitis C, 108 persons who spontaneously cleared the virus, and 588 persons in the control group)	*HLA-DRB1*∗*03:01:01* and *HLA-DRB1*∗*13:01:01*	Persistent HCV infection	[100]
			*HLA-DRB1*∗*04:01:01*, *DRB1*∗*04:05:01*, *DRB1*∗*07:01:01*, and *DRB1*∗*11:01:01*	HCV clearance	

## Data Availability

The data availability is not declared. Our manuscript is a review paper.
